# Acute Mental Health Needs Duration during Major Disasters: A Phenomenological Experience of Disaster Psychiatric Assistance Teams (DPATs) in Japan

**DOI:** 10.3390/ijerph17051530

**Published:** 2020-02-27

**Authors:** Sho Takahashi, Yoshifumi Takagi, Yasuhisa Fukuo, Tetsuaki Arai, Michiko Watari, Hirokazu Tachikawa

**Affiliations:** 1 Department of Disaster and Community Psychiatry, Faculty of Medicine, University of Tsukuba; Tsukuba, Ibaraki 305-8577, Japan; 2 Department of Community and Disaster Assistance, Ibaraki Prefectural Medical Center of Psychiatry, Asahi-machi, Kasama, Ibaraki 309-1717, Japan; 3 Nihon Fukushi University, Okuda, Mihama-cho, Chita-gun, Aichi 470-3295, Japan; 4 DPAT secretariat, commissioned by the Ministry of Health, Labor and Welfare, Kasumigaseki Chiyoda-ku, Tokyo 100-8916, Japan; 5 Kanagawa Psychiatric Center, Serigaya, Konan Ward, Yokohama, Kanagawa 233-0006, Japan; 6 Department of Psychiatry, Division of Clinical Medicine, Faculty of Medicine, University of Tsukuba, Tsukuba, Ibaraki 305-8577, Japan

**Keywords:** disaster, Kumamoto earthquake, DMHISS, disaster psychiatry, Japan, acute mental health needs, duration of activity, DPAT (Disaster Psychiatric Assistance Team)

## Abstract

Background: How long acute mental health needs continue after the disaster are problems which must be addressed in the treatment of victims. The aim of this study is to determine victims’ needs by examining activity data from Disaster Psychiatric Assistance Teams (DPATs) in Japan. Methods: Data from four disasters were extracted from the disaster mental health information support system (DMHISS) database, and the transition of the number of consultations and the activity period were examined. Results: Common to all four disasters, the number of consultations increased rapidly from 0–2 days, reaching a peak within about a week. The partial correlation coefficient between the number of days of activity and the maximum number of victims showed significance. The number of victims and days of activity can be used to obtain a regression curve. Conclusions: This is the first report to reveal that mental health needs are the greatest in the hyper-acute stage, and the need for consultation and the duration of needs depends on the number of victims.

## 1. Introduction

In recent years, the question of how to respond to psychiatric disorders, as well as psychological problems, that arise among disaster victims has become a significant challenge. Many investigative studies have shown that the incidence of disorders such as post-traumatic stress disorder (PTSD) and depression increase markedly after disasters [1]. The criteria for PTSD require that the person was exposed to actual or threatened death, serious injury, or sexual violence [2]. There is a relationship between PTSD and disaster response. It is also known that psychosocial problems that accompany disasters, such as poverty, community breakdown, and the exacerbation of pre-existing psychiatric disorders, have long-term effects on the well-being of victims. Furthermore, existing psychiatric hospitals and health centers in disaster areas lose functionality during disasters and become unable to adequately undertake their consultations and diagnostic functions.

Following recent disasters around the world, such as Hurricane Katrina, the 9/11 terrorist attacks, and major earthquakes, postdisaster mental health services (MHS) have been organized and various kinds of mental health support have been provided [3]. The Inter-Agency Standing Committee (IASC), a nongovernmental organization (NGO), established a set of guidelines in 2007 on how mental health support should be conducted during disasters. These guidelines position mental health support for disaster areas within the broader context of psychosocial support, to create a system of combined mental health services and psychosocial support services (MHPSS), with interventions divided into four levels of specialized services, namely, focused specialized services, nonspecialized support, community and family support, and basic services and security [4]. Concerning specialized services, the guidelines state that interventions by specialists in psychiatry or psychology are necessary, but that due to the diverse social and cultural backgrounds of people in disaster areas, it is not possible to provide guidelines with regard to the specific services to be offered or the types of organized activities to be carried out.

Japan has been significantly affected by disasters, with one quarter of global disasters having occurred there in recent decades [5]. In the Hanshin-Awaji Earthquake of 1995, many people suffered from PTSD, and a psychiatric aid station was established to provide assistance. This led to a broader awareness of the need for psychological support for disaster victims. The 2011 East Japan Earthquake caused many people to suffer from PTSD due to the tsunami, along with the considerable stress suffered by those living in evacuation shelters. In response, several organizations and institutions, such as universities with psychiatry departments, academic associations, and NGOs organized disaster medical services [6], involving the creation of mental health care (*kokoro no care* in Japanese) teams, which carried out support work in the disaster areas. However, a lack of coordination among the teams, as well as their varied composition, led to confusion when providing assistance. There were also tragic cases in which patients in psychiatric hospitals were left behind during evacuation procedures immediately after the disaster, and who died because they could not be brought to the evacuation sites.

Drawing on these experiences, the DMAT system was modified to take into account specific psychiatric needs, and officially approved disaster psychiatric assistance teams (DPATs), which are dispatched to provide mental health support services to people in disaster areas, were established in 2013. The Ministry of Health, Labor, and Welfare (MHLW) determined the definition and role of a DPAT, set up a DPAT head office in Tokyo, and established DPATs in each prefecture and in designated cities in Japan. Since then, the number of teams across the country has gradually increased to 374 in 2017. Meanwhile, the support activities of DPATs have expanded as various disasters have occurred, such as volcanic eruptions, landslides, floods, and earthquakes. We found no other reports of such teams that have been organized to respond to mental health needs in acute phases of disasters in such a specialized and sophisticated manner. 

However, clear guidelines have still not been determined for DPATs concerning the scale of activities to be carried out and at what point the work should be concluded. Because *kokoro no care* team that was active before the creation of DPATs focused primarily on mid- to long-term psychological support, data cannot be obtained that would contribute to the development of practical guidelines for acute phase activities. In relation to DPATs, a disaster mental health information support system (DMHISS) and an online database where all teams document their consultation activities have been established and are used in support activities, and it is now possible to do comparative studies using shared disaster support activity data. Therefore, we have examined data on support activities from multiple disasters that have occurred since the creation of DPATs. The aims of this study were to consider: (1) the scope of mental health needs in the acute phase of disasters, (2) whether organized MHS composed of specialists is necessary, and (3) the scale and time period necessary for the activities of acute-phase MHS.

## 2. Materials and Methods

### 2.1. Subject of Investigation

The period of investigation extended from 2013, when the DPAT system was first created, until 2016. The subject of investigation concerned the activity reports of DPATs, as well as daily reports from the DMHISS database, in relation to four disasters where support activities were conducted during that period, namely, the eruption of Mount Ontake (2014), the Hiroshima landslides (2014), the Kanto-Tohoku torrential rains (2015), and the Kumamoto earthquake (2016).

In the 2012fiscal year, the Toshiba Solutions Corporation was commissioned by the MHLW to develop a disaster mental health information support center. This resulted in the creation of the DMHISS, an online information system that aims to consolidate the activity logs of MHS and contribute to support that is appropriate for the needs of specific disaster areas. It came into nationwide operation in March 2013. 

The functions of the DMHISS are divided to cover: (1) a normal period (prior registration of support teams), (2) an initial response period (coordination of support teams to be dispatched), (3) an activity period (collection of activity logs), and (4) a postactivity period (processing and analysis of activity logs). To facilitate these functions, an activity log collection tool allows data to be divided into microdata to record individual consultation trends and to record the daily activity logs of each DPAT team.

Items to be entered into the activity logs include team information (for example, team name, affiliation, and dispatch period) and type of support (for example, the results of processed data). Before and after its period of activity, each DPAT team enters its activity records into the DMHISS microdata sheet and the daily activity log. As a result, information concerning the people receiving consultations and details about the support activities of that day can be shared in real time between the disaster area itself, the base headquarters, the coordination headquarters, and the DPAT head office, thus making it possible to acquire detailed information about the activities of each DPAT [7].

### 2.2. Methods of Investigation

The definition of a DPAT, the structure of the organization, and an overview of its activities are first set out, followed with an explanation of the methods of investigation.

#### 2.2.1. The Definition of a DPAT, the Structure of the Organization, and an Overview of Its Activities

A DPAT is a psychiatric team that has received specialized training for disaster management, which enables it to be dispatched to disaster areas. Additionally, DPATs are organized according to the administrative districts responsible for responding to disasters.

**Figure 1:**
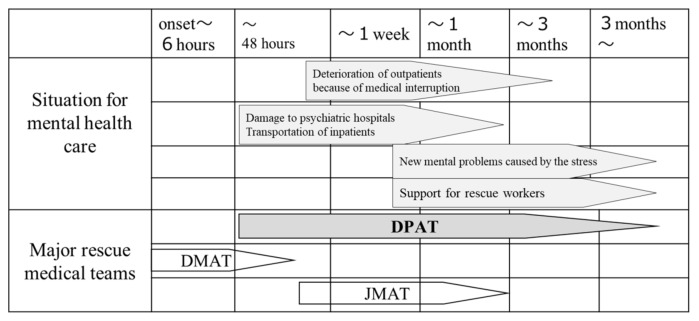
Postdisaster mental health care needs and DPAT dispatch periods. JMAT: Japan medical association team, DMAT; Disaster medical assistance team, DPAT; Disaster psychiatric assistance team.

**Figure 2:**
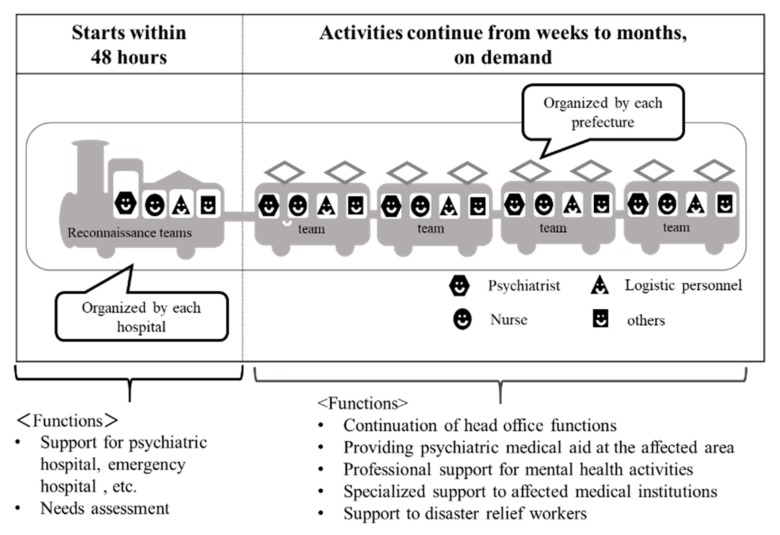
The roles of the reconnaissance Disaster Psychiatric Assistance Team (DPAT) and subsequent DPAT teams.

**Figure 3:**
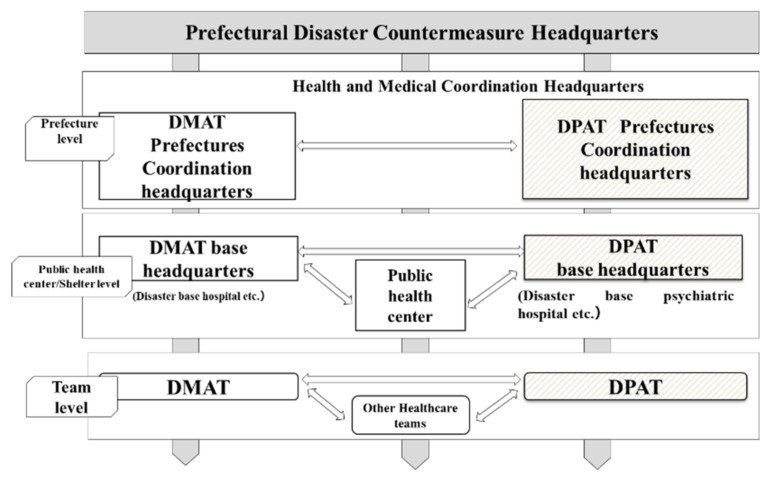
The Disaster Medical Assistance Team (DMAT) operational system.

The main activities of a DPAT are: (1) responding directly or indirectly to the psychological problems experienced by residents in a disaster area at evacuation shelters, (2) coordination of the transport of patients from psychiatric hospitals whose functionality has been affected by the disaster, and (3) support for disaster relief workers. When DPATs are called into action, they move quickly to the disaster area or accident site, i.e., within 48 h, and in coordination with government agencies, fire departments, police departments, and defense forces, they conduct support activities for up to several months until there is no further need (Figure 1). A DPAT team consists of five people (composed of one psychiatrist, two nurses, and two logistics personnel for administrative work). Each team is dispatched to a site to conduct support activities for approximately one week. A reconnaissance team arrives in the disaster area within 48 h, sets up a DPAT coordination headquarters, coordinates with DMATs, coordinates the dispatched teams, and provides initial services at evacuation shelters. After that, the regional DPATs that follow continue the support activities in one-week rotations (Figure 2). The DPATs engage with the disaster countermeasure headquarters operating at the prefectural level, as well as with appropriate authorities at the health center and evacuation shelter levels, and at the level of each team, and coordinate with the DMATs to determine a plan for conducting support activities. Under the direction of each DPAT headquarters, each DPAT can carry out its support activities at evacuation shelters and elsewhere in a coordinated and integrated fashion (Figure 3). Under the instructions of the disaster coordinator, DPATs also coordinate with medical teams such as DMATs, while medical teams coordinate with police, fire departments, and defense forces; health workers coordinate with health care centers.

DPATs arrive slightly later than DMATs, entering the disaster area within 48 h, and continue their activities for longer than DMATs, depending on the scale of the disaster. The timeline of their activities remains unspecified. DPAT: Disaster Psychiatric Assistance Team; DMAT: Disaster Medical Assistance Team; JMAT: Japanese Medical Association Team.

The reconnaissance team enters the disaster area within 48 h, establishes a headquarters, provides support for affected psychiatric hospitals and other health care institutions as required, and conducts need assessments for the disaster area. The subsequent teams provide services such as mental health care assistance, psychological support, and support for disaster relief workers in the affected area. These subsequent teams operate on a rotational basis.

DPATs engage with the disaster countermeasure headquarters at the prefectural level, with appropriate authorities at the health center and evacuation shelter levels, and at the team level with other teams, and collaborate with DMATs to determine a plan for support activities. Under the direction of the DPAT headquarters, each DPAT team carries out its support activities at evacuation shelters and elsewhere in a coordinated and integrated fashion.

#### 2.2.2. Methods of Investigation

First, we summarized the damage situation and DPAT activities in relation to the four aforementioned disasters based on activity reports.

Next, we extracted data from the DMHISS database for these disasters, and created a data set for the daily reports. Following this, we aggregated the information on the dispatch and support structures for each disaster area, changes over time in the number of consultations provided, the number of teams engaged in support activities, and the duration of the period of activity. Furthermore, we divided the dispatch and support structures into four models as proposed by Katō et al. [8]: (a) an outside support model, wherein multiple support workers come from outside the disaster area to provide assistance; (b) a supervision model, wherein outside support workers serve as supervisors, but the support activities are carried out by local staff; (c) a local collaboration model within the region, wherein local organizations such as mental health and welfare centers take the lead in providing support, and; (d) a general support model, wherein mental health is incorporated into ordinary health care services.

We also estimated the duration period of activity for DPATs. At the time of estimation, we supplemented our data with data concerning the support provided by mental health care (*kokoro no kea*) teams before the creation of the DPAT system in relation to the Sayō-chō floods, the Niigata Chūetsu earthquake, the Hanshin-Awaji earthquake, and the East Japan earthquake, as derived from studies published in various documents such as the Cabinet Office’s White Papers on Disaster Management and interviews conducted in each disaster area.

Concerning ethical considerations, we used only anonymous aggregated data from the DMHISS and publicly available documents for this study. This study was carried out with the approval of the Japan Psychiatric Hospitals Association Ethics Committee (approval number 161110-01).

## 3. Results

### 3.1. Comparison of Dispatch Systems for the Four Disasters

We compared the characteristics of the four disasters and the DPAT dispatch systems (Table 1). The disasters were of diverse types, comprising a volcanic eruption, flood disasters, and an earthquake. Although the number of casualties varied, in terms of damage conditions in relation to the number of people evacuated, the numbers of casualties steadily increased, from low numbers with the volcanic eruption, then rising for the flood disasters, and peaking with the earthquake.

In the acute phase, DPAT dispatch and support operated in accordance with the local collaboration model within the region for the Mount Ontake disaster, the supervision model for the Hiroshima and Kanto-Tohoku disasters, and the outside support model for Kumamoto disaster; that is, the greater the scale of the disaster, the more outside support it required. In the mid- to long-term period, Hiroshima and Jōsō moved to a local collaboration model within the region, while Kumamoto transitioned to a supervision model. The period for which teams were dispatched varied a great deal, from 6 days to 3 months. In terms of the total number of teams dispatched, Mount Ontake had the smallest number of teams dispatched, i.e., three teams, while Kumamoto had the highest number, with 1242 teams taking part in support activities. In terms of the number of consultations, there were a total of only 12 for Mount Ontake, but over 100 each for Hiroshima and Jōsō, and 2125 for Kumamoto. In other words, the greater the scale of the disaster and the greater the number of evacuees, the greater the number of teams that were dispatched, and the longer the period of support activity.

### 3.2. Changes over Time in the Number of Consultations in the Four Disaster Areas

Next, we compared changes over time for the number of support consultations in each of the four disasters.

**Figure 4:**
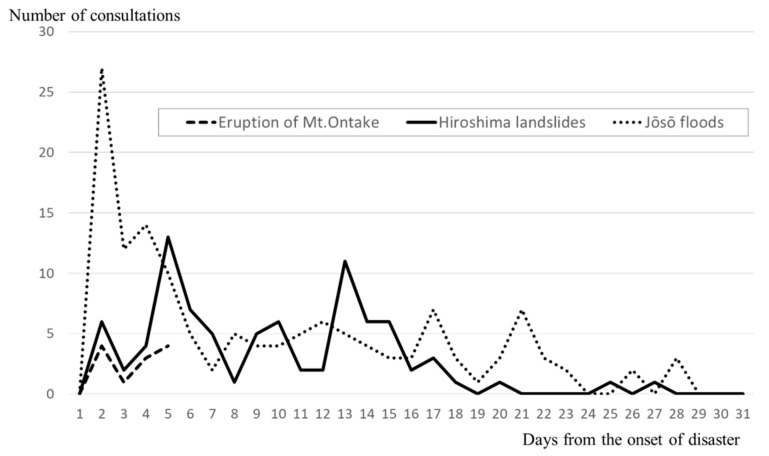
Changes over time in the number of consultations per day for the Mount Ontake eruption, the Hiroshima landslides, and the Jōsō floods.

Figure 4 shows the changes in the number of consultations over time for three localized disasters, i.e., Mount Ontake, Hiroshima, and Jōsō. In the case of the eruption of Mount Ontake, the period of assistance lasted only four days, since many of the victims were people who had come from other prefectures for sightseeing and who were sent back to their home regions after four days of assistance. For the Hiroshima landslides, the number of consultations per day peaked on the 4th day, and on the 12th day after the flooding. After these days, the number of consultations gradually decreased, reaching 0 after approximately 1 month. For the Jōsō floods, the highest number of consultations occurred on the 1st day, at 27, then hovered in the 10–20 range for the next 3 days, after which it decreased to 7 consultations on the 16th and 20th days, before converging to 0 after approximately 1 month.

The vertical axis shows the number of consultations per day, while the horizontal axis shows the number of days following the disaster. The lines of the graph represent the changes in the number of consultations over time for each disaster.

**Figure 5:**
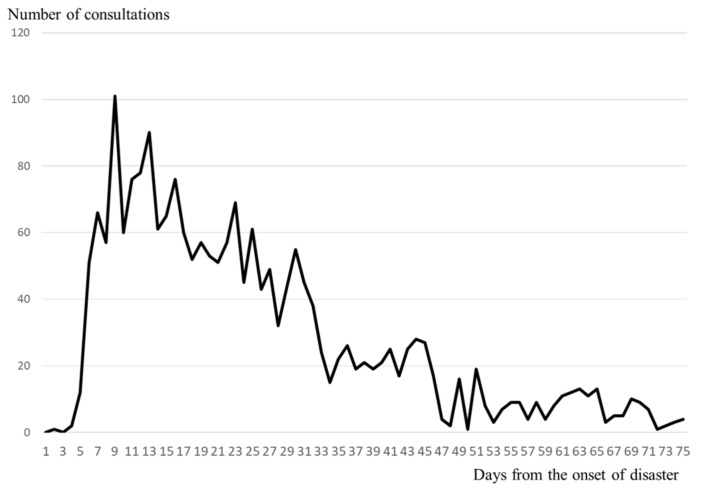
Change over time in the correspondence count per day for the Kumamoto earthquake.

Figure 5 shows the changes in the number of consultations over time for the Kumamoto earthquake. Immediately after the earthquake, patients were moved out of psychiatric hospitals that were at risk of collapsing, so support consultations at evacuation shelters essentially started on the 5th day. There were 66 consultations on the 6th day after the earthquake, with the number peaking on the 8th day, with 101 consultations. After that, the number of consultations decreased with fluctuations, but there were still more than 20 consultations on the 45th day. Two months after the earthquake, the number finally decreased to 15, and assistance continued with fluctuations before concluding after a total of 2–3 months.

The vertical axis shows the number of consultations per day, while the horizontal axis shows the number of days following the disaster.

For all four disasters, the number of consultations started to increase substantially on days 0–2, and reached a peak within approximately 1 week (1–8 days); the larger the scale of the disaster, the longer it took for the number of consultations to converge to 0. Especially in Kumamoto, the scale of the disaster was significantly greater than for the other three disasters, and the number of consultations also showed greater multimodal variation.

### 3.3. Estimation of the Period of DPAT Support from the Scale of the Disaster

In order to quantitatively consider the relationship between the scale of the disaster and the duration period of support, we also investigated the duration of the support period, the maximum number of evacuees, the number of dead, and the number of buildings damaged for other large-scale disasters in Japan, and compared the results (Table 2). An investigation of the partial correlation for each index showed that the partial correlation coefficient between the number of days of assistance and the maximum number of evacuees was significantly high, at 0.97 (*p* < 0.001).

**Figure 6:**
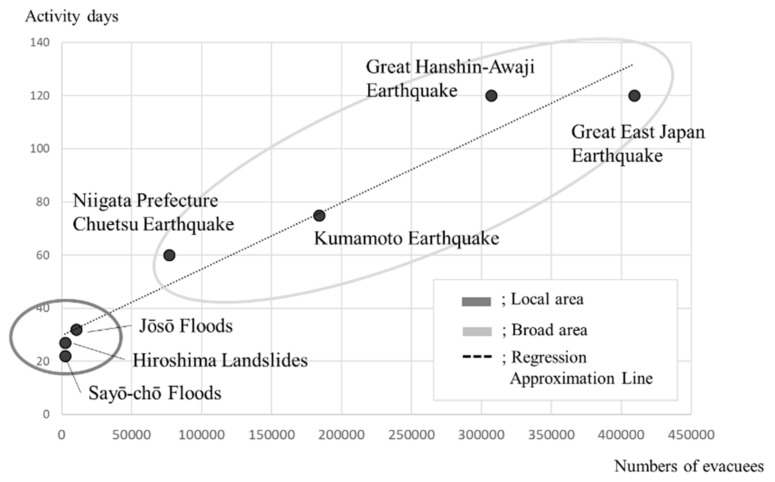
Plot of the team activity days and the maximum number of evacuees.

A scatter plot detailing the number of evacuees and the number of days of assistance, where the number of evacuees is *x* and the number of days of assistance is *y*, yielded a regressive curve of *y* = 0.0002*x* + 29.797 (R2 = 0.95) (Figure 6).

Figure 6 plots the disasters on a scatter diagram, where the vertical axis represents the team activity period (number of days) and the horizontal axis represents the maximum number of evacuees (number of people). The dotted line represents the regression line, the numerical formula represents the regression line formula and the regression coefficient, and the ellipses show the concentrations of localized disasters (Sayō-chō, Hiroshima, and Jōsō) and large-scale disasters (Niigata, Kumamoto, Hanshin-Awaji, and East Japan).

## 4. Discussion

Using mainly DPAT activity data, this study elucidated the need for mental health care in the acute phase of a disaster, the systems necessary to provide it, and criteria for determining the duration of team activity. This study is novel, not only because it provides an outline of the assistance activities of DPATs for the first time using real-time and objective data, but also in the sense that it has determined indices not seen in reports thus far to relate mental health needs in the acute phase of a disaster, the duration period of activities typically carried out in MHS in the acute phase, and the scale of the disaster. 

In the section below, we provide a summary and interpretation of the results of the investigation.

### 4.1. The Role of DPATs Nationally and Internationally

In the MHPSS teams that existed prior to the DPAT system, mental health needs in the acute phase were not gauged, there was not a clear chain of command, and there was no training for support workers, so various problems tended to arise and were less effectively addressed in disaster areas. It would appear, when considering that in the Jōsō floods the DPATs successfully conducted assistance activities in coordination with the Japan Red Cross, and that in the Kumamoto earthquake, patients were successfully transported in a safe and organized manner, that the DPAT system has proved itself, in comparison to the MHPSS, to be a useful disaster mental health service in Japan, especially in the acute phase.

In the United States, under the assistance of the Federal Emergency Management Agency (FEMA) [9], the administrative agencies of each state have jurisdiction over various disaster support activities. However, although various psychological first aid (PFA) guidelines have been established, there are no specialized and standardized teams for disaster psychiatry. For example, mass shooting episodes have increased in recent decades with crisis response teams, which is not for disasters [10]. In Thailand, the Department of Mental Health established a mental health crisis assessment and treatment team (MCATT) system in 2012 [11]. A MCATT comprises a team of specialists including psychiatrists, doctors, psychiatric nurses, nurses, psychologists, and social workers, to provide assistance to disaster victims; it works in coordination with medical emergency response teams, disaster medical assistance teams, and surveillance rapid response teams. According to official requirements, ministries must configure subdistrict or state-level MCATTs with at least one team in each regional hospital (HEALTH, 2018). In South Korea, Korean disaster psychiatric assistance teams (K-DPATs) have begun activities [12] based on the DPAT system in Japan. However, any results concerning the activities of these teams remain unreported.

Compared with these other international MHPSS systems, a unique characteristic of the DPAT system is that standardized teams, centered around a psychiatrist and which have received specialized training, carry out organized mental health care activities starting from the ultra-acute phase after a disaster. Through sharing the results of DPAT activities both in- and out- side of Japan, this system has the potential to become a leading model in disaster mental health care.

### 4.2. Mental Health Care Needs in the Acute Phase of a Disaster

The number of consultations increased immediately after the disaster, reaching a peak in the ultra-acute to acute phase (ranging from 2 to 7 days), regardless of the disaster. Since psychological reactions result from the physical danger experienced immediately following a disaster, and since the most common conditions that arise in a disaster such as PTSD and depression are diagnosed after a certain period of observation, there has been a conventional general belief that mental health support may best be carried out after the acute phase. However, the results of this study have shown that mental health care needs are highest in the ultra-acute phase, so it is necessary for DPATs to do everything they can during this period. Based on the results of the responses to the four disasters, we also noted that consultation needs depended heavily on factors related to the scale of the disaster, such as the number of evacuees. For DPAT activities henceforth, it would be advisable to conduct overall preparation and training to provide swift and large-scale assistance starting from the ultra-acute phase and proportionate to the number of evacuees.

### 4.3. The Duration Period of DPAT Activities

The required duration period of DPAT activities can be determined by the time it takes for a local area’s mental health and welfare system to recover. However, opinions vary as to what constitutes “recovery”, and DPATs have struggled from their inception with the question of when to conclude their activities. Based on the regression equation for the two-dimensional scatter plot, *y* = 0.0002*x* + 29.797, with approximately one month as the y-intercept, we estimate that the recovery period increased by two days for each 10.000-maximum number of evacuees. Further, the same formula suggested that disasters should be considered separately, as both localized disasters, wherein assistance activities may last no more than one month, and disasters that occurred over a wide area, wherein assistance activities may last more than two months, had recovery times which were substantially influenced by the number of evacuees. The use of this kind of predictive formula for determining the duration period of activity has not been reported by DMSs working elsewhere. Furthermore, considering that in the mid- to long-term effects, the support model tended to change to a close collaboration model within the affected region, the basic period of DPAT support should perhaps be set at 1 month as a standard, and a method for smoothly transferring responsibilities back to the local mental health and welfare system should be considered. Furthermore, the periods of time following a disaster are usually divided into the immediate aftermath (ultra-acute phase), the acute phase, the middle phase, and the recovery phase (the long-term), but the number of days comprising each period has not been clearly defined. Considering our results, we suggest that, to facilitate recovery management, the time periods may be defined as an ultra-acute phase lasting 1–2 weeks, an acute phase lasting 1 month, and a mid- to long-term phase for subsequent engagement. As acute-phase mental health care assistance activities by DPATs and other forms of assistance continue to expand in the future, the predictive formula and its interpretation developed in this study should provide practical guidance for recovery interventions.

### 4.4. Limitations of this Study

This study had various limitations. Only a limited amount of data could be obtained concerning disasters, and there was a lack of prior relevant research. We searched the literature for intervention and comparative studies, but could not find any matches. Also, there was no study like this, so comparisons could not be made. There are only 12 literature publications, but there are few comparable studies, which was a difficult point. A search of past disaster dispatched psychiatric teams for all years showed no results. Few countries have done this, and few have reported on their activities; that is why there are few cited references. Therefore, we think that this paper has novel points.

More data on disasters should be accumulated. Also, the analysis concerning the duration period of activity included recent major disasters, but the MHPSS system was responsible for the mid- to long-term recovery period in relation to support activities conducted following the Hanshin-Awaji Earthquake and the East Japan Earthquake; hence, strictly speaking, its activities cannot be compared with those of the DPATs. In future, further data should be accumulated to aid in the undertaking of more precise analyses.

## 5. Conclusions

1. We compared activity data from four disasters where DPATs had provided support.
2. We discovered that mental health care needs were greatest in the ultra-acute phase following a disaster.
3. We discovered that the duration of the activity period was 1 month for localized disasters, or longer for disasters covering a wide area, depending on the number of evacuees.
4. It is necessary to accumulate more cases and examples to ensure more precise analyses.

## Figures and Tables

**Table 1:** Aggregated data on damage situations and DPAT activities for the four disasters.

	Mt. Ontake Eruption	Hiroshima Landslides	Jōsō Floods	Kumamoto Earthquake
Onset date	2014/9/27	2014/8/20	2015/9/9	2016/4/14
Type of disaster	Eruption	Flood	Flood	Earthquake
Maximum evacuees	-	2354	6223	183,882
The number of dead	58	74	2	267
Model (early phase)	c	b	b	a
Model (middle and long term)	-	c	c	b
DPATs dispatch period (days)	6	23	27	90
Total number of DPATs	3	43	28	1242
Total number of consulting cases	12	106	139	2125
Sex (male/female/unknown)	5/7/0	43/63/0	58/70/11	717/1357/51

a–d: due to Models of disaster assistance [8]. Models of disaster assistance. (a) Outside support model: in which many support personnel come from outside the disaster area and provide support. (b) Supervision model: in which external support personnel act as supervisors, with support activities undertaken by regional staff. (c) Local collaboration model: in which the staff of mental health welfare centers mainly comprise locals. (d) General support model: in which mental health support is incorporated into ordinary health care services.

**Table 2:** The number of DMS activity days and major indicators of damages in large-scale disasters.

Name of Disaster	Team Activity Days	Maximum Number of Evacuees	The Number of Dead	Number of Houses Damage
Sayō-chō Floods	22	2219	20	1790
Hiroshima landslides	27	2257	58	4559
Jōsō Floods	32	10,390	2	8327
Niigata Prefecture Chuetsu Earthquake	60	76,615	68	123,664
Kumamoto Earthquake	75	183,882	50	206,148
Great Hanshin-Awaji Earthquake	120	307,022	6400	256,312
Great East Japan Earthquake	120	409,146	15,735	1,137,785
Partial correlation coefficient (R2) with team activity days	-	0.97	−0.01	−0.57
